# Fluoxetine-mediated inhibition of endoplasmic reticulum stress is involved in the neuroprotective effects of Parkinson’s disease

**DOI:** 10.18632/aging.101716

**Published:** 2018-12-24

**Authors:** Tao Peng, Xiaoyan Liu, Jingtao Wang, Yu Liu, Zhenqiang FU, Xingrong Ma, Junmin Li, Guifang Sun, Yangfei Ji, Jingjing Lu, Wencui Wan, Hong Lu

**Affiliations:** 1 Key-Disciplines Laboratory Clinical Medicine Henan, Department of Neurology, the First Affiliated Hospital of Zhengzhou University, Zhengzhou 450052, Henan Province, China; 2 Department of Ophthalmology, the First Affiliated Hospital of Zhengzhou University, Zhengzhou 450052, Henan Province, China

**Keywords:** fluoxetine (FLX), Parkinson's disease (PD), neurons, X‑box‑binding protein 1 (XBP1), caspase-3

## Abstract

Background: Accumulating evidence suggests that Fluoxetine (FLX), an anti-depressant drug, has broad neurobiological functions and neuroprotective effects in central nervous system injury, but its roles in Parkinson's disease (PD) remain unclear. In this study, we aimed to evaluate whether fluoxetine attenuates rotenone-induced neurodegeneration in PD.

Methods: Male Sprague-Dawley rats were randomly allocated to control, rotenone-treated, rotenone + FLX-treated and FLX-treated groups. Behavioral tests including open field behavioral test and catalepsy measurement were taken to evaluate neurological behavioral measurements. Apoptosis was detected by TUNEL assay. Endoplasmic reticulum (ER)-related gene expressions were detected by qRT-PCR and western blot. Immunohistochemistry was performed to assess dopaminergic neuronal degeneration.

Results: We demonstrated that pretreatment with FLX (10.0 mg/kg, i.p.) significantly ameliorated the catalepsy symptom and increased locomotor activity. In addition, FLX markedly reversed the loss of dopaminergic neurons and suppressed the X‑box‑binding protein 1 (XBP1)/caspase-3-activated ER stress. Furthermore, FLX inhibited rotenone-mediated neurodegeneration through caspase-3-mediated neuronal apoptosis.

Conclusion: Taken together, our findings indicate that FLX has beneficial neuroprotective effects in PD and FLX might be a potential therapeutic agent for the treatment of PD. In light of its favorable properties, FLX should be evaluated in the treatment of PD as well as related neurologic disorders.

## Introduction

Parkinson's disease (PD) is the second most common degenerative disorder of the central nervous system which is characterized with cell death of dopaminergic (DA) neurons in the brain’s basal ganglia worldwide [1–3]. Currently, there is no cure for PD, with treatment directed at improving symptoms. Although researches have been performed, the pathophysiology of PD is still unknown.

Endoplasmic reticulum (ER) stress is involved in the pathogenesis of PD [4–6]. ER plays a key role in protein synthesis, glycosylation, and folding [7–9]. Moreover, evidence showed that ER stress could be induced by rotenone in vitro and in vivo [10]. Previously studies proved that ER stress could lead to cell death by activating cysteine aspartic proteinase-3 (caspase-3)-mediated apoptotic pathway, and CHOP was identified to be the crucial member in the ER stress inducing apoptosis [11–13]. Fluoxetine (FLX) is a selective serotonin reuptake inhibitor functions as an antidepressant and exerts neuroprotective effects via its anti-inflammatory effects [14–16]. However, it is yet to be clarified whether FLX affects the degeneration and PD behavior in the rotenone rat model of PD. In this study, we sought to investigate whether FLX attenuates rotenone-induced neurodegeneration in PD rats through suppression of ER stress pathway.

## RESULTS

### FLX promotes the motor activity of PD rats

**Figure 1:**
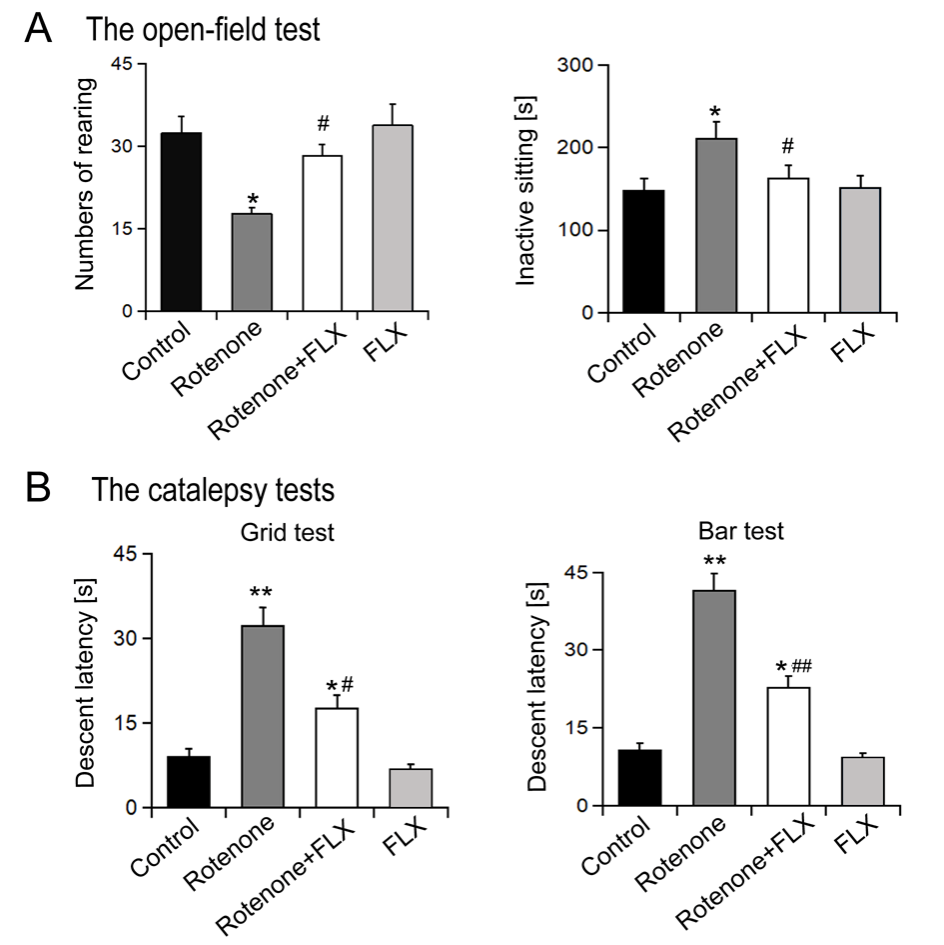
**Effects of FLX treatment on degradation of motor activity of PD rats.** (**A**) Open field behavioral test, n=10 each group. (**B**) Catalepsy measurement, n=10 each group. Data were presented as mean ± SD, *P<0.05, **P<0.01 versus control group; #P<0.05, ##P<0.01 versus rotenone-treated group.

As shown in Figure 1A, the open field test results showed that comparing to the control group, rotenone treatment reduced the numbers of rearing and prolong the period of inactive sitting, whereas combined treatment with FLX reversed these results (P < 0.05). Catalepsy tests showed that chronic rotenone treatment resulted in a significantly prolonged descent latency compared with the control group (P < 0.001, Figure 1b). The catalepsy induced by rotenone was markedly antagonized with FLX treatment (P <0.001, Figure 1B).

### FLX prevents DA depletion in the striatum of PD rats

**Figure 2:**
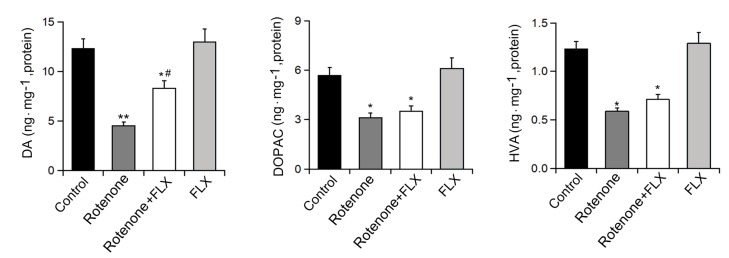
**Effects of FLX treatment on DA depletion in Striatum of PD rats.** Striatal DA, DOPAC, and HVA were measured by HPLC, n=6 each group. Data were presented as mean ± SD, *P<0.05, **P<0.01 versus control group; # P <0.05 versus rotenone-treated group.

In contrast to the control group, significantly lower levels of DA, DOPAC, and HVA were observed in rotenone-induced rats. Treatment with FLX could partially recover the decrease of Striatal DA induced by rotenone, but had no significant effect on the levels of DOPAC and HVA (n=6, p<0.05, Figure 2). In addition, treatment alone with FLX did not change the levels of DA and metabolites in rats (n=6, P<0.05, Figure 2).

### FLX prevents the decrease of DA neurons in PD rats

**Figure 3:**
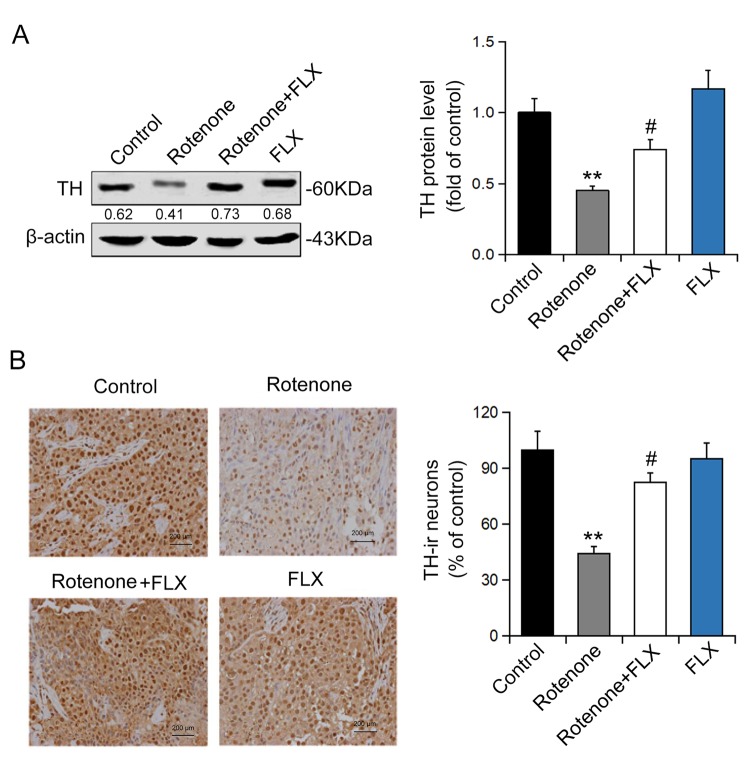
**Effects of FLX on DA neurons loss in PD rats.** (**A**) TH protein expression was detected using Western blot, n=6. (**B**) TH-positive DA neurons were determined using immunohistochemistry. The ratio of TH-positive neurons in experimental groups to those cells in the control group was evaluated. n=4, **P<0.01 versus control group; #P<0.05 versus rotenone-treated group.

To detect the effect of FLX on DA neurons we evaluated the expression of TH using Western blot and immunohistochemistry. The results showed that rotenone treatment significantly decreased the expression of TH protein and this effect was abolished by further treatment with FLX (n = 6, P <0.05, Figure 3A). In addition, the number of TH-positive neurons was reduced following rotenone treatment, whereas treatment with FLX reversed this result (n = 6, P <0.05, Figure 3B).

### FLX inhibits ER stress in PD rats

**Figure 4:**
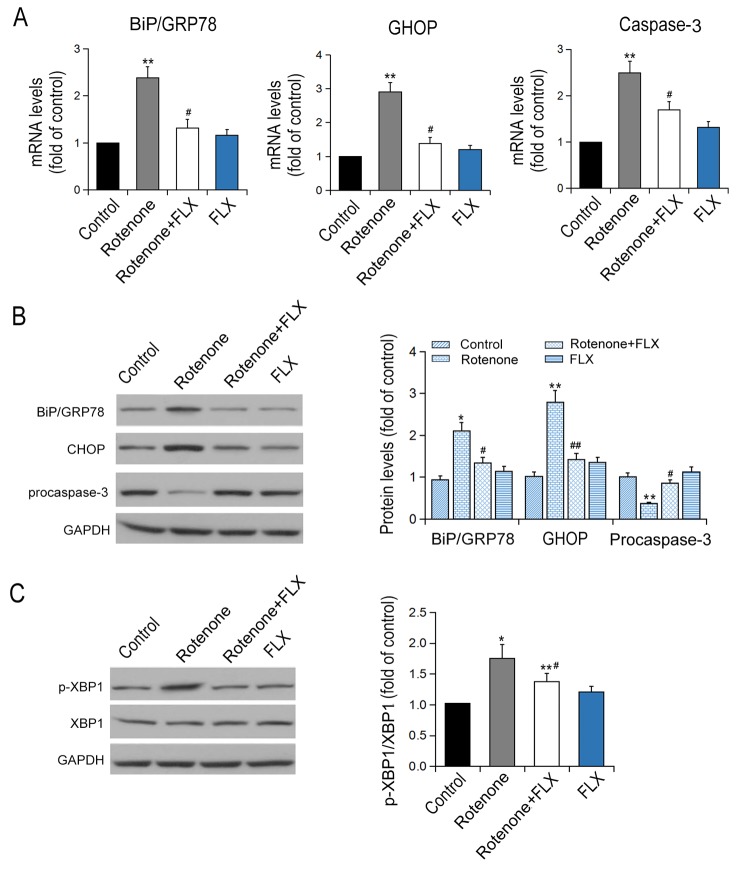
**Effects of FLX on ER stress in the brain of PD rats.** (**A**) Transcriptional levels of Bip/GRP78, CHOP and caspase-3 were measured by RT-qPCR, n=6. (**B**-**C**) Translational levels of Bip/GRP78, p-XBP1, XBP1, CHOP and procaspase-3 were detected using Western blot, n=6 each group. *P<0.05, **P<0.01 versus control group; # P <0.05, ##P<0.01 versus rotenone-treated group.

Rotenone-treated rats were found to have remarkably increased transcriptional and expressional levels of BiP, CHOP, and caspase-3 compared with controls (P < 0.001, Figure 4A). Furthermore, PD rats treated with FLX displayed a considerable decrease in mRNA levels of BIP, CHOP, and caspase-3 (P <0.001, Figure 4A). Similar changes in protein expression levels of BIP and CHOP were confirmed by Western blot (P <0.001, Figure 4B). Additionally, rotenone treatment reduced procaspase-3 expression and this effect was abolished by FLX treatment (P <0.001, Figure 4B). Furthermore, rotenone treatment increased the phosphorylation of XBP1 protein at ser724 site, which indicates the activation of XBP1, while FLX treatment diminished the phosphorylation of XBP1 (P <0.001, Figure 4C). Together, these data indicate that FLX inhibits ER stress induced by rotenone.

### FLX suppresses the apoptosis of DA neurons induced by ER stress in PD rats

**Figure 5:**
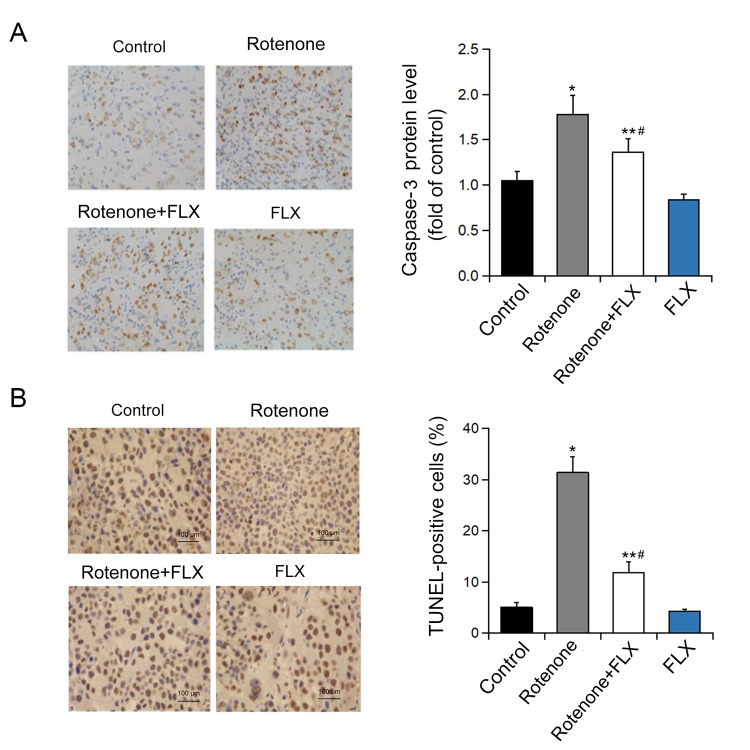
**Effects of FLX on ER stress-mediated neuron apoptosis in PD rats.** (**A**) Caspase-3 protein level was determined by immunohistochemistry. n=4. (**B**) Quantification of TUNEL-positive cells. *P<0.05, **P <0.001 versus control group, #P <0.001 versus rotenone-treated group. Scale bar = 100 μm.

**Figure 6:**
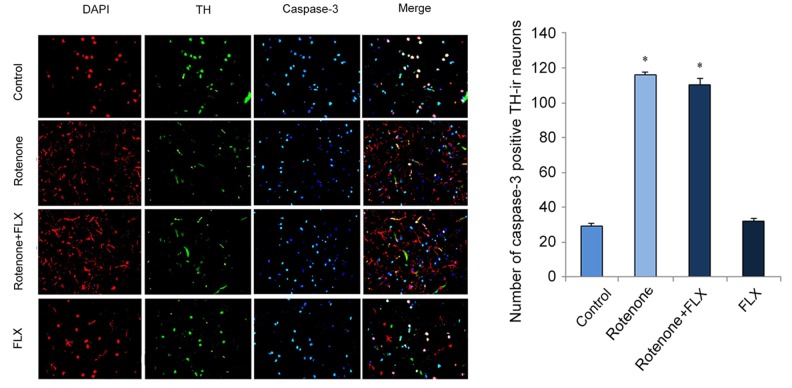
**Effects of FLX on caspase-3 expression in DA neurons of PD rats.** Anti-TH (red) and anti-caspase-3 (green) were used in dual immunostaining examination. Cell nuclei were stained with DAPI (blue).

Caspase-3 was significantly activated in brain of rotenone-induced PD rats, while the activation of caspase-3 was diminished in PD rats treated with FLX (P < 0.001, Figure 5A). In addition, treatment with FLX at least partially reverse in the effect of rotenone on the apoptosis of DA neurons as characterized by TUNEL analysis (P < 0.001, Figure 5B). Furthermore, the reversal effect of FLX on rotenone-induced caspase-3 expression in DA neurons was confirmed by dual immunostaining. In contrast to control rats in which caspase-3 was only weakly stained, rotenone induced a strong staining of caspase-3 protein in DA neurons. Moreover, FLX treatment weakened those caspase-3 positive staining remarkably (Figure 6).

## DISCUSSION

Clinical and genetic toxicology studies proved that ER stress is involved in the pathogenesis of PD [17]. Previously research showed that higher levels of phosphorylated PERK and eIF2α were observed in PD rats [18]. Another study showed that the expression of UPR and the phosphorylation of XBP-1, p-Erk, and eIF2α were found to be increased in MN9D cell line when treated with 6-OHDA and MPP [19]. It is well-known that BIP and CHOP are the characteristics of ER stress and the high levels of these protein will induce ER stress [20]. As one of the main ER stress regulators, CHOP contributes to ER stress-mediated cell apoptosis via depressing bcl-2 promoter and decreasing bcl-2 expression [21]. Here, we showed that FLX had neuroprotective effects on rotenone-induced PD rats as characterized by the improvement of ethology and biochemical pathology in PD rats. This is the first study to prove the protection of FLX which was functioned via inhibiting XBP1-caspase-3 mediated ER stress.

FLX shows neuroprotective effects in a rat model of cerebral ischemia, it was demonstrated that FLX reduced the extent of brain injury and improved functional outcomes [22]. Here, our present data suggested that TH immune-staining could be replaced with DA detection for quantitation of DA neuronal degeneration.

In addition, our study showed that rotenone significantly upregulated the transcriptional and translational levels of BIP and CHOP, which suggested chronic treatment of rotenone led to a persistent and severe ER stress. And FLX inhibited ER stress by suppressing the expression of BIP and CHOP. Meanwhile, rotenone stimulated the increase of XBP1 phosphorylation. It has been proved that caspase-3 is located in ER stress-specific caspase cascade and activated in ER stress-induced apoptosis rather than mitochondria-mediated apoptosis [23,24]. In this study, dual immunostaining examination showed that, in contrast to the control rats, rotenone induced a strong staining of caspase-3 protein in DA neurons of PD rats. This revealed that caspase-3 mediated apoptosis was involved in DA neuronal degeneration. Besides, FLX treatment weakened caspase-3 positive staining remarkably and decreased neuronal apoptosis, which meant FLX had an anti-apoptosis effect. In a word, these results indicated that caspase-3 was involved in ER stress-mediated apoptosis, and FLX suppressed rotenone-induced ER stress-specific apoptosis. Finally, previous researches proved that XBP1 activated caspase-3 and c-Jun N-terminal kinase (JNK) to induce apoptosis. Meanwhile, our data revealed that XBP1 phosphorylation was closely related with caspase-3 activation. This suggested that FLX inhibited ER stress via suppressing activation of XBP1/caspase-3 pathway and played a neuroprotective role in rotenone-induced PD rats.

## CONCLUSION

In summary, in this study, we revealed that FLX had neuroprotective effects on rotenone-induced PD rats. And this is the first time that FLX is proved to prohibit ER stress via repressing the activation of XBP1/caspase-3 pathway.

## MATERIALS AND METHODS

### Reagents and preparation

Rotenone (Sigma Aldrich, Saint Louis, Missouri, USA) was emulsified in 2.5 mg/ml sunflower oil and FLX was dissolved in 30% DMSO/saline for injection. Microsyringe pump (Alzet Corporation, Palo Alto, CA) was incubated in 0.9% aseptic brine at 37°C for overnight.

### Animal management

Male Sprague-Dawley rats (200-220 g, 12-14 weeks old) were purchased from Shanghai laboratory animal center of the Chinese Academy of Sciences. All experimental procedures were in accordance with the guidelines of the Laboratory Animal Manual of the National Institute of Health Guide to the Care and Use of Animals, which were approved by the Biological Research Ethics Committee of the First Affiliated Hospital of Zhengzhou University.

### Experimental groups

Rats were randomly allocated to control, rotenone-treated, rotenone + FLX-treated and FLX-treated groups (n=6). Sunflower oil + 30% DMSO (diluted with saline infusion), rotenone + 30% DMSO, rotenone + FLX and sunflower oil + FLX were injected to control group, rotenone-treated group, rotenone + FLX-treated group and FLX-treated group, separately. Rotenone and sunflower oil were injected intraperitoneally with 1ml/kg/day for 6 weeks. FLX was injected one hour before rotenone injection through intraperitoneal (i.p.) injection with 10 mg/kg/day for 15 consecutive days. The dose for FLX was determined according to a previous study from Podurgiel SJ et al. [25].

### Open field behavioral test

We used a square plastic box with inside walls painted black. The animals were placed into the center of the square and allowed to move freely for 5 min. After rotenone treatment for 42 days, the behaviors of rats were recorded using video-tracking system, including the times of standing-up and sitting quietly as previously described [26].

### Catalepsy measurement

Catalepsy measurement contained two different tests. The first test was determined by gently placing four paws of the rat on a vertical grid. The intensity was estimated by counting the time the animal moved one paw from the grid with a maximal “cut-off” of 120 s. The second test was measured by gently placing both forepaws of the rat on the top of a metal stick. The intensity was assessed by counting the time the animal climbed down from the stick with a maximal “cut-off” of 120 s as previously described [27].

### HPLC analysis

Striatal DA, DOPAC and HVA were analyzed by high performance liquid chromatography (HPLC) coupled to electrochemical detection (ED). The striatum was removed and homogenized in 0.1 M perchloric acid using ultrasonic homogenizer. The samples were determined using UV detection and the mobile phase comprised 0.1 M citrate buffer, 0.02 mM EDTA, 1 mM octane sulfonate and 10% methanol was delivered at a flow rate of 1 ml/min. Data were analyzed using Breeze software (version 3.2, Waters, Massachusetts, USA).

### qRT-PCR

Total RNA was extracted from frozen brain tissues using TRIzol reagent (TaKaRa, Dalian, China) according to the manufacturer’s instructions. The reverse transcription (RT) reactions were performed using a Prime Script™ RT Reagent Kit (Takara). qRT-PCR was performed on Applied Bio systems Inc 1900 using SYBR Green Real Time PCR Kit (Bio-Rad, America). The reaction is as follows: 95°C 30 seconds, then 95°C 10 seconds, 55°C 45 seconds for 40 loops. The relative expression of each target gene was calculated by using 2-ΔΔCT method standardized to GAPDH.

### Western blot

Total proteins from brain tissues were extracted and separated on 15% SDS-PAGE gels. Then proteins were transferred onto PVDF and subsequently blocked in 5% non-fat milk for 2 h at room temperature. Membranes were immunoblotted with the indicated primary antibodies: BiP, caspase-3 (1:800; Santa Cruz Biotechnology, Santa Cruz, CA, USA), XBP1 (1:1000; Novus Biologicals, MO, USA), Tyrosine Hydroxylase (TH, 1:800; Santa Cruz Biotechnology) and β-actin (1:1000; Santa Cruz Biotechnology) overnight at 4°C. After washed with TBST, the membranes were incubated with HRP-conjugated secondary antibody (1:2000; Sigma-Aldrich, USA) for 2 h at room temperature. Finally, the proteins were visualized using enhanced chemiluminescence detection and quantified by densitometry using Image J software. β-actin was used for the internal control.

### Immunohistochemistry

Brain tissues were extracted and fixed with PEA solution overnight at 4°C. After slicing from the brain at a distance of 24-26 mm from anterior fontanelle, the tissues were paraffin embedded and cut into 5 μm coronal sections according to the standard protocol. Then after dewaxing and hydration, the sections were treated with 0.5% Triton X-100 for 30 min and subsequently 5% BSA for another 30 min. The slices were immunoblotted with the indicated primary antibodies: caspase-3 (1:100; Santa Cruz Biotechnology) and XBP1 (1:2000; Sigma-Aldrich) overnight at 4°C. Furthermore, the sections were incubated with secondary antibody (1:1000; Sigma-Aldrich) for 1 h at room temperature. Finally, the slices were quantified using GEDA-801D image detection system and visualized by fluorescence microscopy (Olympus, Tokyo, Japan).

### TUNEL apoptosis detection

Apoptosis was detected using In Situ Cell Death Detection Kit (POD; Roche,USA). After dewaxing and hydration, the sections were incubated with proteinase K for 20 minutes at room temperature. Then the slices were washed with PBS and treated with mixture of TdT and nucleic acid for 1 h at 37°C. Subsequently, the slices were counterstained with hematoxylin (Sigma-Aldrich.) and dehydrated. The final observation was performed by microscopy.

### Statistical analysis

All data were presented as the mean ± standard deviation (SD). Statistical significance was assessed by ANOVA and Tukey’s test in SPSS 20.0 statistical software (Chicago, Illinois, USA). P < 0.05 was considered statistically significant.
